# The Production of Meningococcus Anti-Endotoxin

**Published:** 1918-11

**Authors:** 


***The Production of Meningococcus Anti-Endotoxin.*** By M. H. Gordon. *The British Medical Journal,* Sept. 28, 1918.

In the case of antimeningococcus serum, the highest clinical potency is not obtained unless the serum has a definite amount of anti-endotoxin. Meningococcus anti-endotoxin is by no means easy to obtain, since at present only occasional batches of anti-meningococcus serum are found to contain it in any quantity. Nevertheless, it would seem to be very desirable that antimeningococcus serum should possess as high a value as possible in regard to anti-endotoxin.

*Procedure.* Young rabbits of a thousand to two thousand grams weight were used, and all the injections were made intravenously. The antigens compared have been (1) the raw coccus suspended in saline; (2) the same coccus after being sensitized by leaving it all night in contact with a good specimen of antimeningococcus serum, which is drawn off next morning, and the agglutinated cocci re-suspended in saline; (3) the raw coccus autolyzed by leaving it for three days at 37° C.; (4) the raw coccus softened and partially dissolved by suspending it in N/40 NaOH; (5) a standard suspension

(0.1 gram powder to 5 cc. water) of the dried coccus ground up and suspended in distilled water; (6) a suspension of the dried coccus of similar strength, but with the endotoxin inactivated by leaving it in the incubator over night in contact with N/1 NaOH, subsequently reduced to N/40. All of the above antigens were preserved by the addition of 0.5 o/o of phenol.

It would seem that anti-endotoxin was more readily elaborated by the rabbit to type II meningococcus than to type I. The sensitized coccus and the suspension of dried coccus would appear to be more consistently successful than the other antigens.

In order to obtain anti-endotoxin, especially in the case of type I meningococcus, it is absolutely essential to avoid over dosage.

The antigens that appeared to give best results in the rabbit are neither the raw coccus nor autolysates, but a suspension of the dried coccus, and the sensitized raw coccus.

